# Comparative metagenomics approaches to characterize the soil fungal communities of western coastal region, Saudi Arabia

**DOI:** 10.1371/journal.pone.0185096

**Published:** 2017-09-21

**Authors:** Tarek A. A. Moussa, Hassan S. Al-Zahrani, Omar A. Almaghrabi, Tamer S. Abdelmoneim, Michael P. Fuller

**Affiliations:** 1 Biological Sciences Department, Faculty of Science, King Abdulaziz University, Jeddah, Saudi Arabia; 2 Biological Sciences Department, Faculty of Science, University of Jeddah, Jeddah, Saudi Arabia; 3 Botany and Microbiology Department, Faculty of Science, Cairo University, Giza, Egypt; 4 Department of Agricultural Botany, Faculty of Agriculture, Suez Canal University, Ismailia, Egypt; 5 School of Biological Science, Faculty of Science and Engineering, Plymouth University, Plymouth, United Kingdom; Friedrich Schiller University, GERMANY

## Abstract

A total of 145007 reads were obtained from pyrosequencing for all the 4 samples. The total count ranged from 11,301,014 (Mecca old road) to 23,503,512 bp (Thuwal). A total of 460 fungal species belonging to 133 genera, 58 families, 33 orders, 13 classes and 4 phyla was identified across the four sites. The most abundant phylum at all four sites was Ascomycota followed by Basidiomycota. Four phyla (Ascomycota—99.31%, Basidiomycota—0.59%, Chytridiomycota—0.04%, Glomeromycota—0.03%) were detected in Khulais. Except for Glomeromycota, all phyla were detected at Mecca old road (Ascomycota—74.26%, Basidiomycota—25.71%, Chytridiomycota—0.01%) and Thuwal (Ascomycota—99.59%, Basidiomycota—0.40%, Chytridiomycota—0.002%); while only Ascomycota—90.98% and Basidiomycota—9.01% were detected in Asfan road. At the class level, Sordariomycetes was predominantly observed at Asfan road—59.88%, Khulais—68.26% and Thuwal—94.84%; while Pezizomycetes was dominant at Mecca old road—56.01%, was absent at Asfan road. Agaricomycetes was present only at Mecca old road—25.73%; while Tremellomycetes—5.77%, Malasseizomycetes—2.13% and Microbotryomycetes—1.10% were found only at Asfan road. The phylogenetic trees revealed that clear genus level differences are visible across all the four sites, with an overall predominance of *Thielavia* followed by *Madurella*, *Aspergillus*, and *Gelasinospora*. *Chaetomium* sp., *Aspergillus caespitosus* and *Aspergillus* sp. were found in moderate (Mecca old road and Thuwal) to abundant (Asfan road and Khulais) quantities. *Thielavia* sp., *Thielavia hyalocarpa* and *Madurella* sp. are found in moderate quantities at Khulais and Mecca old road, while in abundant levels at Asfan road and Thuwal. *Fusarium equisati* and *F*. *oxysporum* were detected at Thuwal and Khulais. *Sordaria araneosa* was present at Khulais, while *Malasseiza globosa* species was detected in moderate quantities across all sites except Khulais.

## Introduction

The fungal diversity of soils in Saudi Arabia is little understood with no previous published detailed studies in the literature. As an arid to semi-arid region, it is traditionally accepted that fungal diversity will be low with slow decomposition of plant debris a norm in the moisture deprived soils [1–4]. Traditional studies have characterised Saudi Arabian fungi from fruiting bodies and disease symptoms of natural plant species [5–7]. Recently developed metagenomics techniques have been used to study soil fungal diversity and have been able to rapidly characterise the biodiversity of many soils worldwide [8–11]. However, relatively few metagenomic studies have been used to assist in the characterisation of soils in developing counties [12] and none in Saudi Arabia.

Barcodes of DNA, biochemical markers, and investigation of acyl chain organization in the phospholipids layer likewise give intensive tools to microbial ecology without needing to the ordinary culture [7,13]. Of these techniques, DNA barcoding is an intense tool to recognize species utilizing arrangements gene regions, which are conserved across wide and diverse taxa [14,15]. Genes conservation gives an outline for the structure to allocating groupings to genera and species for examinations the diversity of microbial community [10,16]. Despite, its noticeable quality in evaluating bacterial groups, nrSSU is thought to be excessively monitored for segregating fungal species [15,17] while nrITS and nrLSU have all the earmarks of being the additionally encouraging.

The errand of finding a critical part of yet obscure species has just as of late turned out to be conceivable with the coming DNA high-throughput sequencing of natural samples, which can possibly additionally help information obtaining in biodiversity inquire about [18–22]. The methodology of metagenomics includes distinguishing of various species from ecological specimens utilizing specific genes particularly chosen with the end goal of recognition. These genes are commonly similar ones that are utilized as a part of sample based huge scale DNA barcoding endeavours [23,24], henceforth giving both suitable power of determination for the gathering of intrigue and the most extreme measure of reference groupings accessible from vouchered samples.

In this study, we attempted to decipher the microbiome of various soil samples collected from different sites based on two nrITS regions (ITS1/2 and ITS3/4) [25,26].

## Materials and methods

### Ethics statement

No specific permits were required for the field sampling. The collection locations are not privately-owned or protected in any way. Field sampling was taken from bare soil, didn’t involve plant material from endangered or protected species.

### The study region

All sites chosen for study were public sites, Khulais governorate (90 km north-east Jeddah) (MMKhulais_P.SS), Mecca old road (16 km east Jeddah) (MMMeccaORD_P.SS), Thuwal village (80 km north Jeddah on the coast of the Red Sea) (MMThuwal_P.SS) and Asfan road (60 km East Jeddah) (MMAsfan_P.SS) (Table 1). Sampling sites were assigned coordinates via GPS for ease of relocation (Table 1). Field sampling was taken from bare soil, didn’t involve plant material from endangered or protected species. Soil cores were taken at each site (2 cm diameter and 20 cm deep), and 2, 3 or 4 soil samples were collected for each sampling site depending on the heterogeneity of the site and pooled to provide the location sample. Soils were stored in 50 ml Falcons at -20°C till analysis. Whilst most fungal diversity was expected in the uppermost 10 cm of soil, samples to 20 cm were taken to ensure all possible fungal species were sampled.

**Table 1:** Coordinates of the sampling sites.

Sampling site	Coordinates		Sample code
	N	E	
Khulais 1	22° 08ʹ 54.46"	39° 20ʹ 35.02"	MMKhulais_P.SS
Khulais 2	22° 06ʹ 35.90"	39° 18ʹ 41.80"	
Mecca old road 1	21° 27ʹ 07.30"	39° 36ʹ 09.70"	MMMeccaORD_P.SS
Mecca old road 2	21° 25ʹ 59.44"	39° 38ʹ 28.75"	
Mecca old road 3	21° 24ʹ 57.31"	39° 40ʹ 41.30"	
Thuwal 1	22° 17ʹ 18.43"	39° 06ʹ 59.53"	MMThuwal_P.SS
Thuwal 2	22° 17ʹ 47.64"	39° 06ʹ 48.61"	
Thuwal 3	22° 17ʹ 14.12"	39° 05ʹ 52.43"	
Thuwal 4	22° 17ʹ 29.96"	39° 05ʹ 57.12"	
Asfan road 1	21° 54ʹ 56.87"	39° 20ʹ 09.80"	MMAsfan_P.SS
Asfan road 2	21° 51ʹ 57.70"	39° 24ʹ 58.07"	
Asfan road 3	21° 48ʹ 06.76"	39° 28ʹ 52.57"	

### DNA extraction, PCR, and pyrosequencing

The rules for next-generation sequencing description datasets were described by Nilsson et al. [27] were followed. The extraction of genomic DNA from 8 g of soil subsample after mixing the various samples of each site by MoBio Power soil kit was done. GS-FLX high-throughput encoded amplicon sequencing was used for monitoring the diversity of fungi in the sites under investigation. Construction of libraries were done using combinations of the ITS region tagged primers, as a recommendation for the metagenomics technique [28]. The amplification of genomic DNA was performed using ITS1 (5´-XCTTGGTCATT TAGAGGAAGTAA) and ITS4 (5´-YxxxxxTCCTCCGCTTATTGATA TGC) primers, where X and Y were regarded as the two metagenomics primers (CCTATCCCCTGT GTGCCTTGGCAGTCTCAGT and CCATCTCATCCCTGCGTGTCTCCGACTCAGA) and xxxxx regards as the barcodes for sample identification. PCR reaction was done for each sample under the following conditions: 2 min at 95°C, 25 cycles of 30 s at 95°C, 1 min at 54°C, 2 min at 72°C and final step 10 min at 72°C. The PCR mixture contained one μl of template DNA, 10x buffer 4 μl, dNTP’s (2.5 mM) 1.5 μl, each primer (10 mM) 1.5 μl, MgCl_2_ (50 mM) 4 μl, BSA (10 mg/ml) 0.5 μl and *Taq* polymerase (5 U/μl) 0.4 μl and complete the mixture to 40 μl by adding 25.6 μl of water. Four PCR reactions were done for each sample. PCR products were collected, purified and sequencing-barcodes were integrated via Mega Primers. The length and concentration of amplicon were evaluated, and all amplicon libraries were mixed by equimolar and used for pyrosequencing. Labeled samples were collected and sequenced using the 454 GS FLX titanium System (Applied Biosystems, Nutley, NJ, USA) at Macrogen Inc. (Seoul, Korea).

### Data processing and statistical analysis

The Roche GS FLX software (v 2.9) was used to perform GS FLX data processing. The processing of the image was as follows: first subtract background and normalize the images, second find the active wells on the PicoTiterPlate device, third extract the raw signals for each flow in each active well and fourth write the resulting flow signals into “composite wells format” (CWF) files.

The statistical analyses of the fungal communities were used in each process. As the total number of reads before and after filtration depending on four sites, random subsampling was conducted for statistical data analysis. The species richness was defined with the CD-HIT program at a 99% sequence similarity [29]. The rarefaction curve [30] and diversity indices were calculated by Mothur platform [31].

BLASTN was performed against nr database of all read sequences. These results were filtered by selecting reads which were greater than or equal to 100 bp and had read coverage of minimum 70%.

The alignments were performed using ClustalW with best sequence matches based on BLASTN for constructing the phylogenetic trees. the Seqboot program and maximum likelihood trees were constructed using the PHYLIP package (version 3.69) was used to perform bootstrapping, and tree were exported in Newick format. This tree was then uploaded to iTOL [32] along with the colour file to display counts of reads within genera.

## Results

### Pyrosequencing and sequence analysis

The sequencing statistics are summarized in Table 2. A total of 145,007 reads were obtained from pyrosequencing for all the 4 samples, with an average length of 467 bp. The validated reads after filtration ranged from 18,982 (Mecca old road samples) to 46,070 reads (Thuwal samples). The total count was ranged from 11,301,014 (Mecca old road samples) to 23,503,512 bp (Thuwal samples), the maximum read length ranged from 558–780 bp and the average read length ranged from 452–477 bp. The average GC percentage content ranged from 46–55% (Table 2).

**Table 2:** Overview of the raw sequencing data.

	Khulais	Mecca old road	Thuwal	Asfan road
Number of total reads	28,853	24,954	50,442	40,758
Number of validated reads	21,186	18,982	46,070	39,785
Number of bases	13,484,571	11,301,014	23,503,512	19,470,687
Min. read length	8	8	10	10
Max. read length	780	558	848	767
Average read length	467	452	465	477
Average % GC	52.29	46.26	54.67	55.30

### Estimation of species richness and sampling depth

Species richness of sample obtained from Thuwal was the highest among the four sites, followed by Khulais, Mecca old road and Asfan road samples. However, only the curves representing samples from Asfan road and Mecca old road appear to have reached the horizontal asymptote, suggesting sufficient sampling depth and species coverage from respective locations. The curve representing Khulais sample appears to be reaching the plateau (S1 Fig).

### Taxonomic composition analysis

**Fig 1:**
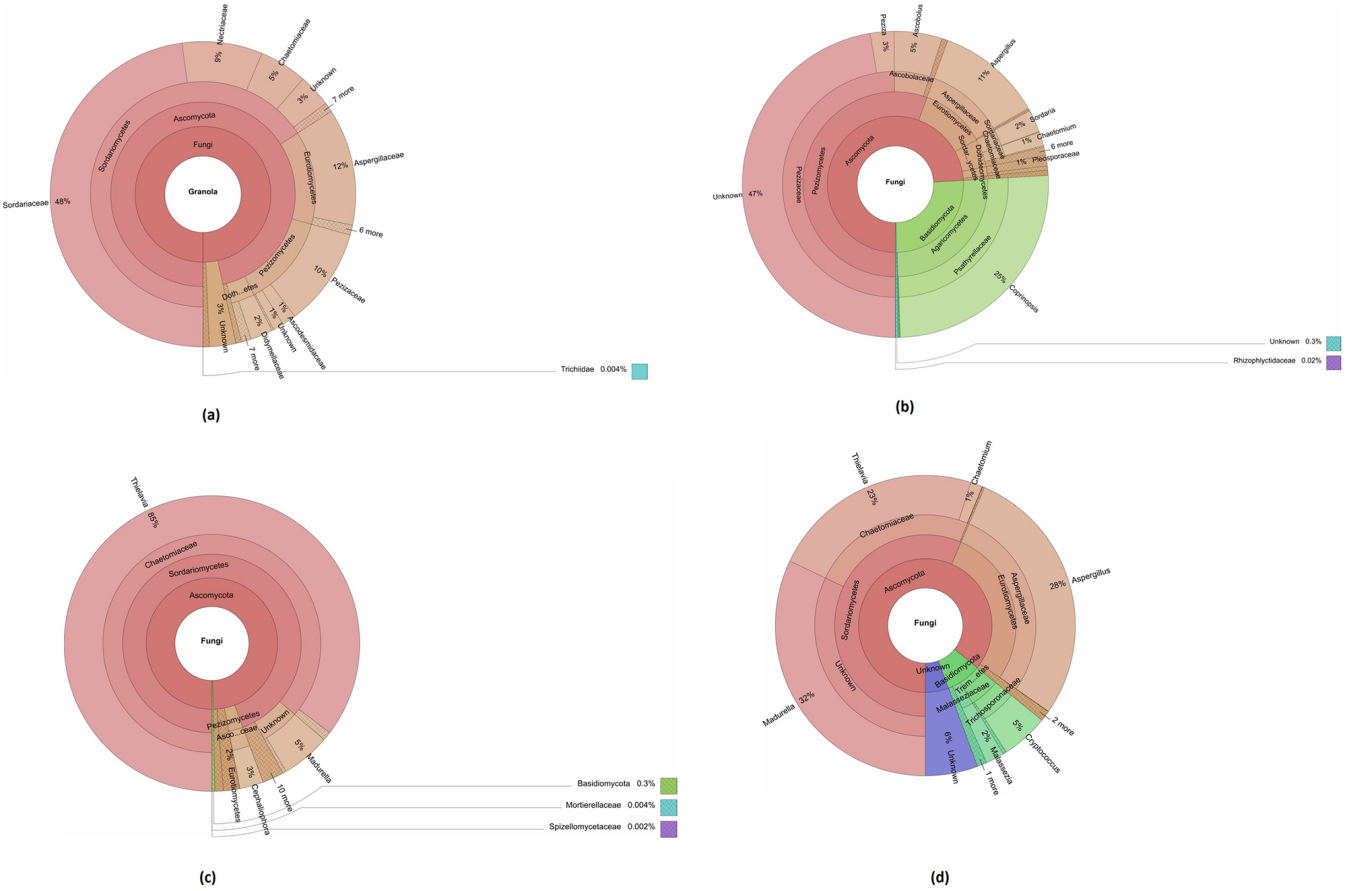
Taxonomic composition of soil samples in the four sites: Khulais (a), Mecca old road (b), Thuwal (c) and Asfan road (d).

Taxonomic hierarchical analysis of each sample was accomplished through generation of Krona plots. Kingdom, phylum, class, family and genus ranks of each sample were selected for representation. Since fungi were dominant in all the samples, only fungi reads were chosen to generate these plots. Less abundant taxa are listed outside the charts along with their relative abundances (Fig 1). The results revealed that the most abundant phylum was Ascomycota in the all four sites followed by phylum Basidiomycota.

### Fungal diversity detection

**Fig 2:**
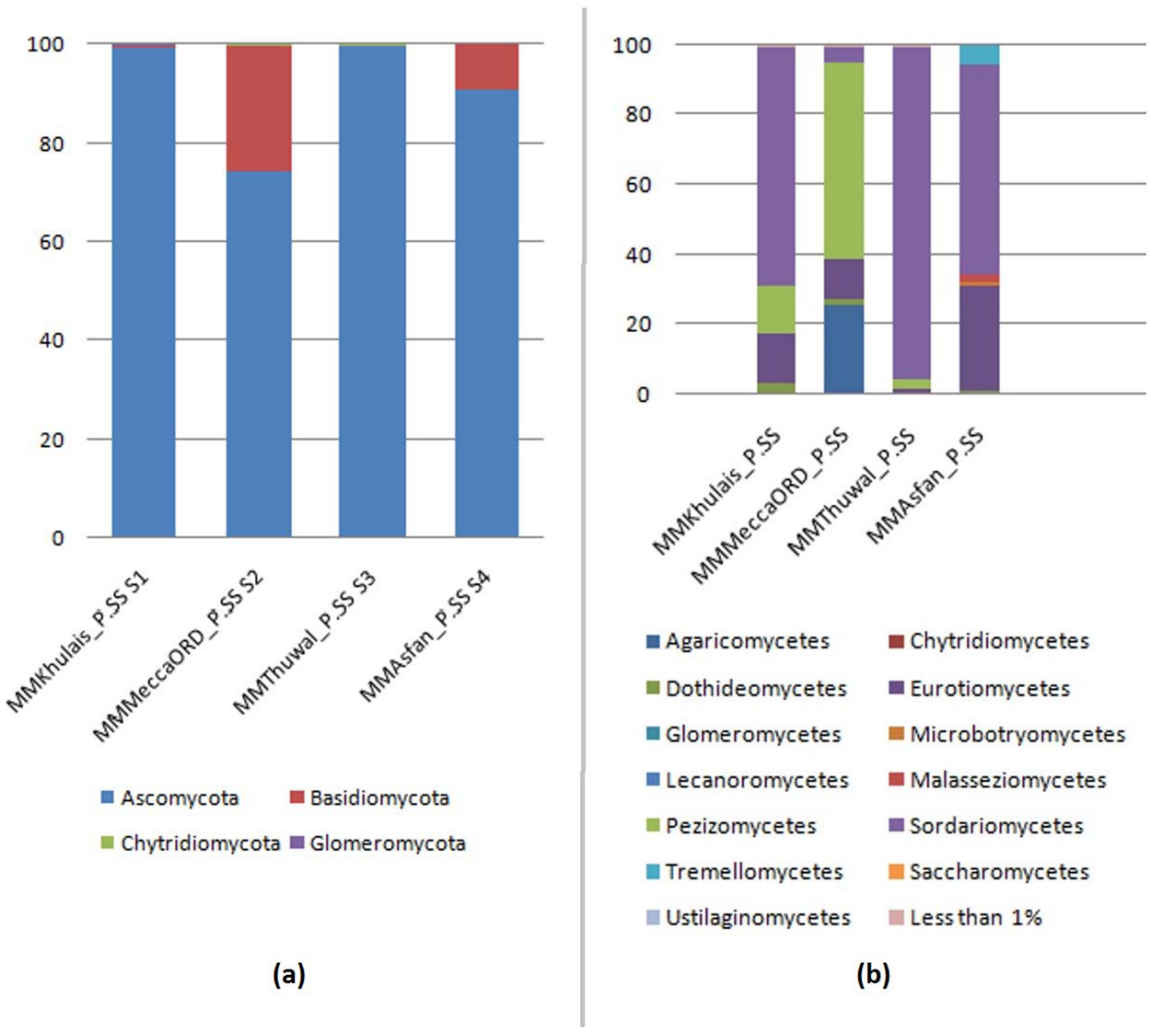
Read distribution of sequences according to phylum (a) and class (b) of fungi in the four sites.

Four phyla (Ascomycota—99.31%, Basidiomycota—0.59%, Chytridiomycota—0.04%, Glomeromycota—0.03%) were detected in samples collected from Khulais. With the exception of Glomeromycota the rest of these phyla were detected in samples from Mecca old road (Ascomycota—74.26%, Basidiomycota—25.71%, Chytridiomycota—0.01%) and Thuwal (Ascomycota—99.59%, Basidiomycota—0.40%, Chytridiomycota—0.002%); while only Ascomycota (90.98%) and Basidiomycota (9.01%) were detected in samples from Asfan road. As evident from the figure, Ascomycota was dominant in all the samples, followed by Basidiomycota (Fig 2a).

The dominant classes were Eurotiomycetes, Sordariomycetes and Pezizomycetes, which showed high differences in the relative abundances across samples. Sordariomycetes was predominantly observed in samples from Asfan road (59.88%), Khulais (68.26%) and Thuwal (94.84%); while Pezizomycetes, which was dominant in sample obtained from Mecca old road samples (56.01%) was absent in Asfan road samples. Class Agaricomycetes was present only in soil samples obtained from Mecca old road (25.73%); while classes Tremellomycetes (5.77%), Malasseizomycetes (2.13%) and Microbotryomycetes (1.10%) were found only in Asfan road samples. Classes that contributed to less than 1% of each sample were grouped separately (Fig 2b).

### Data analysis using rank scoring to evaluate fungal diversity

**Fig 3:**
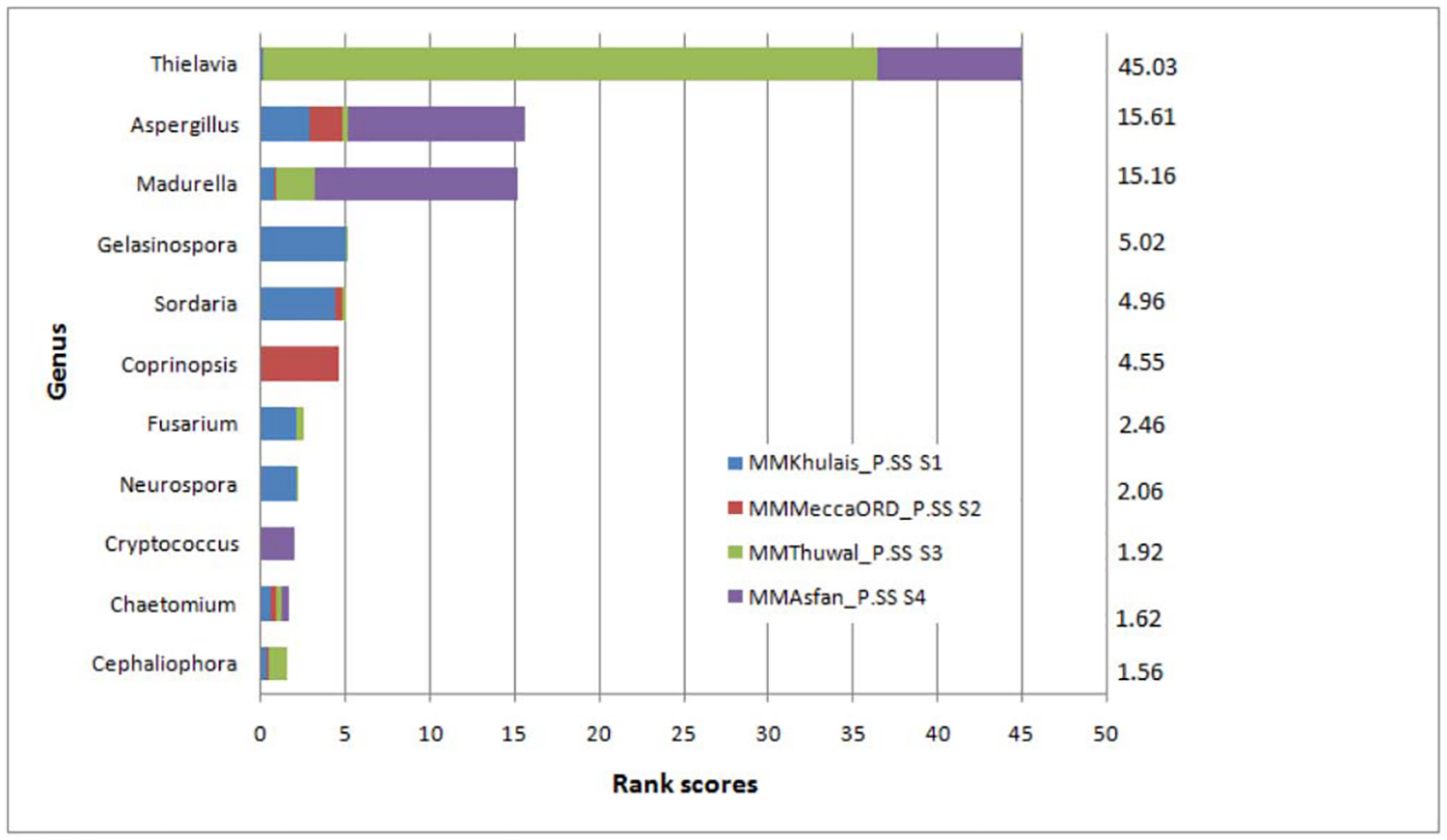
Bar plot of rank scores at genus level.

Only significant genera were considered for calculating rank scores for genera from each sample. The significance of each genus was determined on the basis of its frequency of occurrence in a sample. *Thielavia*, *Aspergillus*, *Madurella* genera were abundantly present at all four sites with high variances in relative abundance among sites; while *Cryptococcus* and *Chaetomium* were detected in moderate to high level at all sites. *Fusarium* was abundantly present in samples from Khulais (2168 reads), in moderate levels in Thuwal samples (458 reads), but absent in Asfan road and Meccan old road samples. *Sordaria* and *Cephaliospora* were detected in abundance at Khulais (4634 reads) and Thuwal (1184 reads) samples respectively, but neither genera were found at Asfan road. *Gelasinospora* and *Coprinopsis* each were abundant at Khulais (5355 reads) and Mecca old road (4825 reads) and in negligent quantities at Thuwal and absent at Asfan road (Fig 3).

### Phylogenetic analysis of fungal community

**Fig 4:**
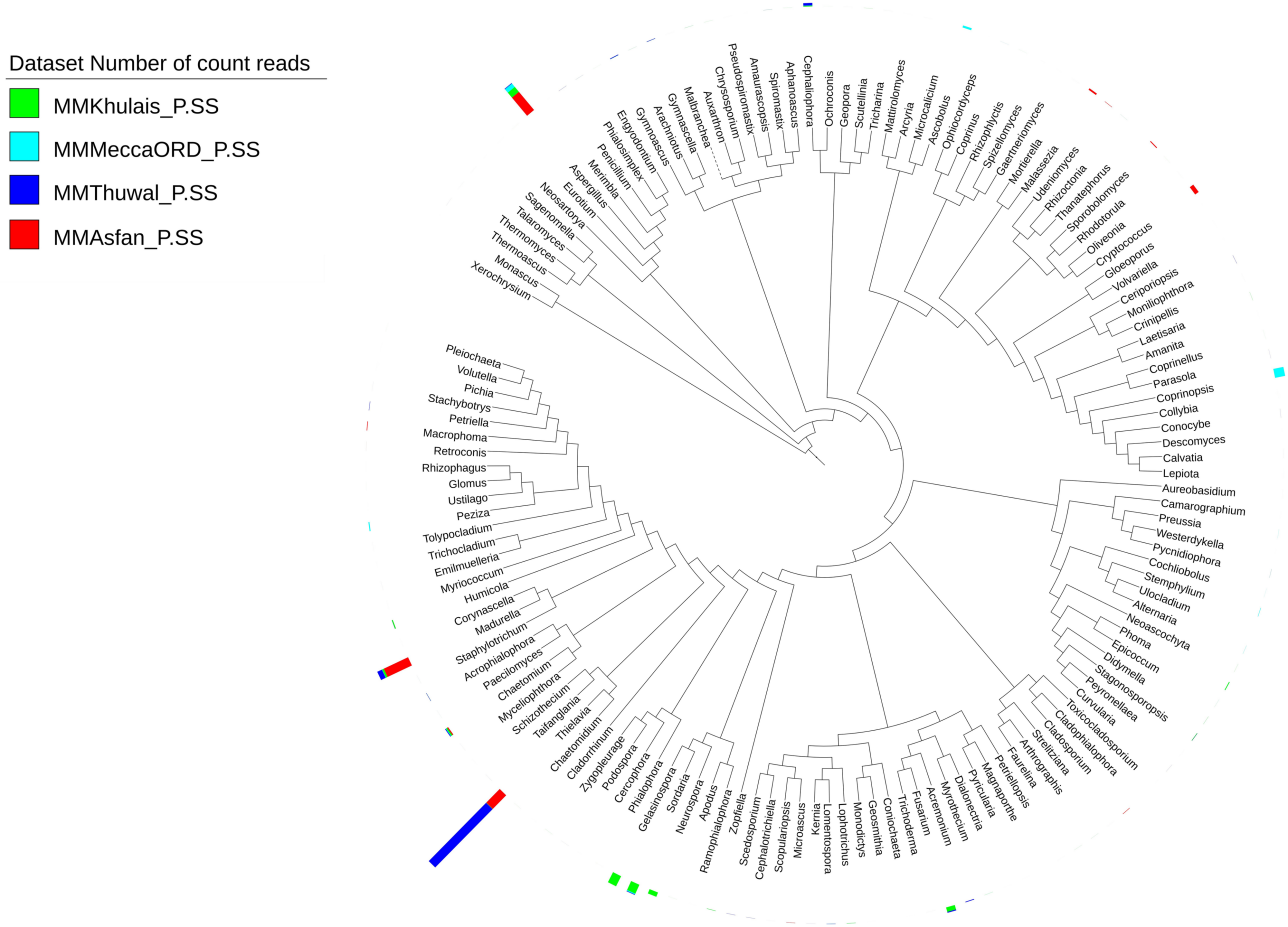
Hierarchical tree representing taxonomic relationships of fungal genera detected in all four sites of Mecca region, Saudi Arabia. The height of the bars in the circle outside the branch tips corresponds to the number of reads within genera. The key to bar color for the samples are at the top right.

Clear genus level differences were visible for all the four sites, with an overall predominance of the genus *Thielavia* followed by *Madurella*, *Aspergillus*, and *Gelasinospora*. The dominant genera were *Thielavia* (at the all four sites with predominance at Thuwal—84.3%), Unknown fungal species belonging to Pezizaceae (at Khulais—10.4% and Mecca old road—47.4%), *Madurella* (at all four sites with predominance at Asfan road—32.1%), *Aspergillus* (at all four sites with predominance at Asfan road—28.25), *Coprinopsis* (at Khulais—0.1% and Mecca old road—25.4%), *Gelasinospora* (at Khulais—21.0%) and *Sordaria* (at Khlais—18.2%, Mecca old road—2.4% and Thuwal—0.5%) (Fig 4).

### Taxonomic heat map analysis of fungal species

**Fig 5:**
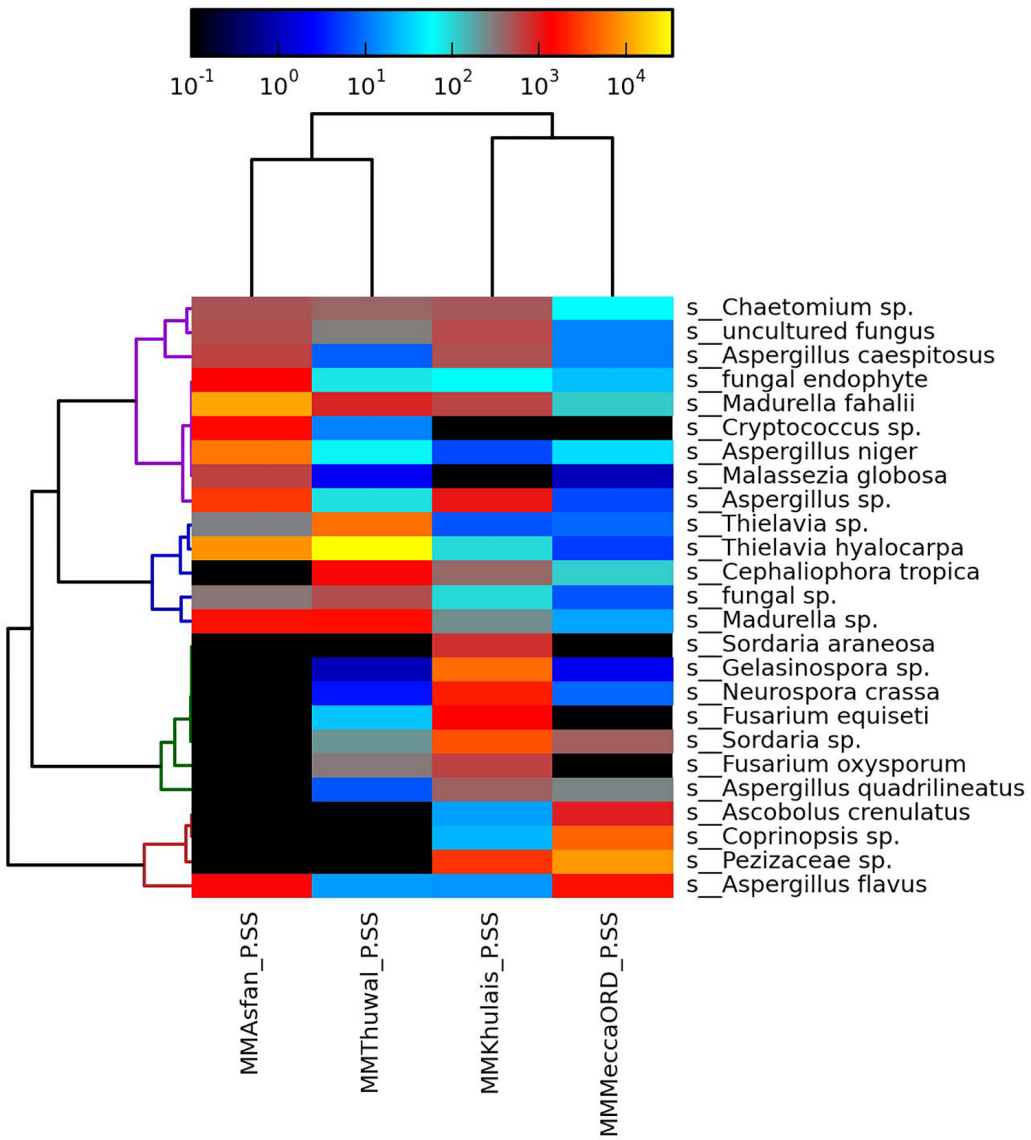
Heat map indicating differences in relative abundances of predominant species in each site.

*Chaetomium* sp., *Aspergillus caespitosus* and *Aspergillus* sp. were found to be in moderate (Mecca old road and Thuwal samples) to abundant (Asfan road and Khulais samples) quantities at all sites (Fig 5). *Thielavia* sp., *Thielavia hyalocarpa* and *Madurella* sp. were found in moderate quantities in Khulais and Mecca old road samples, while in abundant levels in Asfan road and Thuwal samples. *Fusarium equisati* and *Fusarium oxysporum* were detected only in Thuwal and Khulais samples. *Sordaria araneosa* was present only in Khulais samples, while *Malasseiza globosa* species was detected in moderate quantities in all samples except Khulais. Uncultured fungi were detected in high amounts in all the four sites (Fig 5).

### Nucleotide sequence accession number

Metagenome sequence data from this study were submitted to the NCBI Sequence Read Archive (SRA) under accession numbers: SRR3150823 (MMKhulais_P.SS), SRR3144873 (MMMeccaORD_P.SS), SRR3150825 (MMThuwal_P.SS), SRR3150846 (MMAsfan_P.SS). Direct link to deposited data http://www.ncbi.nlm.nih.gov/sra?term=SRP069742

## Discussion

Assessment of fungal diversity in this study focuses on soil communities near the coast of Red Sea due to high importance for nature conservation, recreational purposes and water resources management [33]. The sand dunes of the Coastal region include areas of high ecological diversity as environmental heterogeneity and species structure [34].

All four sites studied were located near the Red Sea coast at Jeddah, Saudi Arabia and a total 145,007 reads were validated based on taxonomic assignment criteria. Rarefaction curves revealed the species richness at the Khulais site had a more diverse fungal community than the other sites, whereas Asfan road samples had the lowest number of species.

The level of fungal group overlap among the soil samples from the known ecosystems and geographical sites was assessed by examining the ITS1 and ITS2 regions. ITS1 and ITS2 sequences were assigned to 1,660 and 1,393 OTUs (based on 97% similarity), respectively [35]. 768 OTUs were analysed from the Ulleungdo samples, while 640 and 382 OTUs were analysed from the Dongdo and Seodo (islets of Dokdo) samples, respectively [36].

In another metagenomic studies, 36 soil samples were obtained over 2 years from four sites. Two of them had an output-decline problem whilst the other two did not. fungal or bacterial were found in these samples More than 2000 OTUs. Relative abundance of each OTU was compared statistically for differences between samples. 721 comparisons were statistically significant containing 366 unique bacterial and 44 unique fungal OTUs [37]. Furthermore, fungal groups in the beds of *Salix repens* showed high diversity and 1211 non-singletons were detected with 97% fungal sequence similarity after analysing 688,434 ITS2 [33].

In the current study, there were two phyla (Ascomycota and Basidiomycota), which were dominant in all sites, Chytridiomycota in three sites and Glomeromycota in one site only. The latter two phyla Chytridiomycota and Glomeromycota were minor with less than 1% abundance. Ascomycota was the most abundant phylum among the four phyla at all four sites. Similarly, Kim et al. [38] detected the five phyla Basidiomycota, Ascomycota, Chytridiomycota, Glomeromycota and Zygomycota in soil zones associated with infection with *Tricholoma matsutake* and found that the fairy ring zone only had three of these phyla (absence of Glomeromycota and Zygomycota) and inside the fairy ring zone only had four (absence of Zygomycota) whereas the Basidiomycota and Ascomycota were dominant in all soil samples tested.

The dominant phylum was Ascomycota (33.18%), the second one was Basidiomycota (22.73%), Glomeromycota (5.29%), Mucoromycotina (1.94%), and Chytridiomycota (0.53%) [33]. Ascomycota and Basidiomycota are the dominant phyla in the soil environment [39–41]. These phyla can be classified into several groups according to their ecological characteristics that is as saprophytic, pathogenic, mycorrhizal, endophytic or lichen-forming fungi. brown-rot fungi, soft-rot fungi, and white-rot fungi are saprophytic fungi and belong to the phylum Basidiomycota. Most the ectomycorrhizal fungi, also belong to the phylum Basidiomycota, which live on the roots of vascular plants [42–44].

Buée et al. [39] concluded that Basidiomycota accounted for 65% and Glomeromycota for 2.24%. In another study, Ascomycota was the most prevalent fungal class, accounting for 36.7 to 93% of all OTUs for most samples from different ecosystems across Italy and France [35].

In the current study, the dominant classes were Eurotiomycetes, Sordariomycetes and Pezizomycetes. Sordariomycetes was predominantly observed in Asfan road, Khulais and Thuwal; while Pezizomycetes was dominant in Mecca old road, was absent at Asfan road. Agaricomycetes was present only at Mecca old road; while Tremellomycetes, Malasseizomycetes and Microbotryomycetes were found only at Asfan road.

The classes of the phylum Ascomycota were commonly found in all soil samples tested, the Leotiomycetes, Eurotiomycetes, unclassified Ascomycetes, Dothideomycetes, Sordariomycetes. At the class level in Basidiomycota, all samples shared Agaricomycetes, unclassified Basidiomycota, Tremellomycetes, Microbotryomycetes [38]. There were two dominant classes of Ascomycota, Eurotiomycetes and Sordariomycetes [45]. In the other study, pyrosequencing analysis of fungal diversity in forest soil revealed that Agaricomycetes was the dominant fungal class [39].

Dothideomycetes, Eurotiomycetes, Sordariomycetes and Agaricomycetes were the dominant classes which though showed high variation in relative abundance across markers [46].

At the genus level, the phylogenetic trees revealed that clear genus level differences were visible at all the four sites, with an overall predominance of the genus *Thielavia* followed by *Madurella*, *Aspergillus*, and *Gelasinospora*. *Chaetomium* sp., *Aspergillus caespitosus* and *Aspergillus* sp. were found in moderate (Mecca old road and Thuwal) to abundant (Asfan road and Khulais) quantities. *Thielavia* sp., *Thielavia hyalocarpa* and *Madurella* sp. were found in moderate quantities at Khulais and Mecca old road, while in abundant levels at Asfan road and Thuwal. *Fusarium equisati* and *Fusarium oxysporum* were detected only at Thuwal and Khulais. *Sordaria araneosa* was present only at Khulais, while *Malasseiza globosa* species was detected in moderate quantities at all sites except Khulais.

At the genus level, it was noticeable that the genus *Aspergillus* and *Schizosaccharomyces* clearly dominate in all the samples examined [47], *Aspergillus* was found in marine sediments [48] and in mangrove sediments [49].

## Conclusion

Metagenomics were a powerful tool for the characterisation of fungal species diversity in soils of Saudi Arabia providing comparative results on many species and genera that would otherwise have taken years of culturing and identification. The soil samples obtained from Asfan road had low species diversity, while those obtained from Khulais and Thuwal had high species diversity. The dominant genera were *Thielavia*, *Madurella*, *Aspergillus* across the all four sites. Uncultured fungi were detected in high amounts across all the four sites. The unknown fungal species were found belonging to the *Pezizaceae* at Khulais and Mecca old road and in the future, we will try to perform functional metagenomics to identify these unknown organisms.

## Supporting information

S1 Fig Species accumulation curves of multiple rarefactions across the four sites. (TIF)
