# Supplementary figures and images for: Comparative metagenomics approaches to characterize the soil fungal communities of western coastal region, Saudi Arabia
Source: PLoS One. 2017 Sep 21;12(9):e0185096. doi: 10.1371/journal.pone.0185096 (PMC5608318; doi:10.1371/journal.pone.0185096)

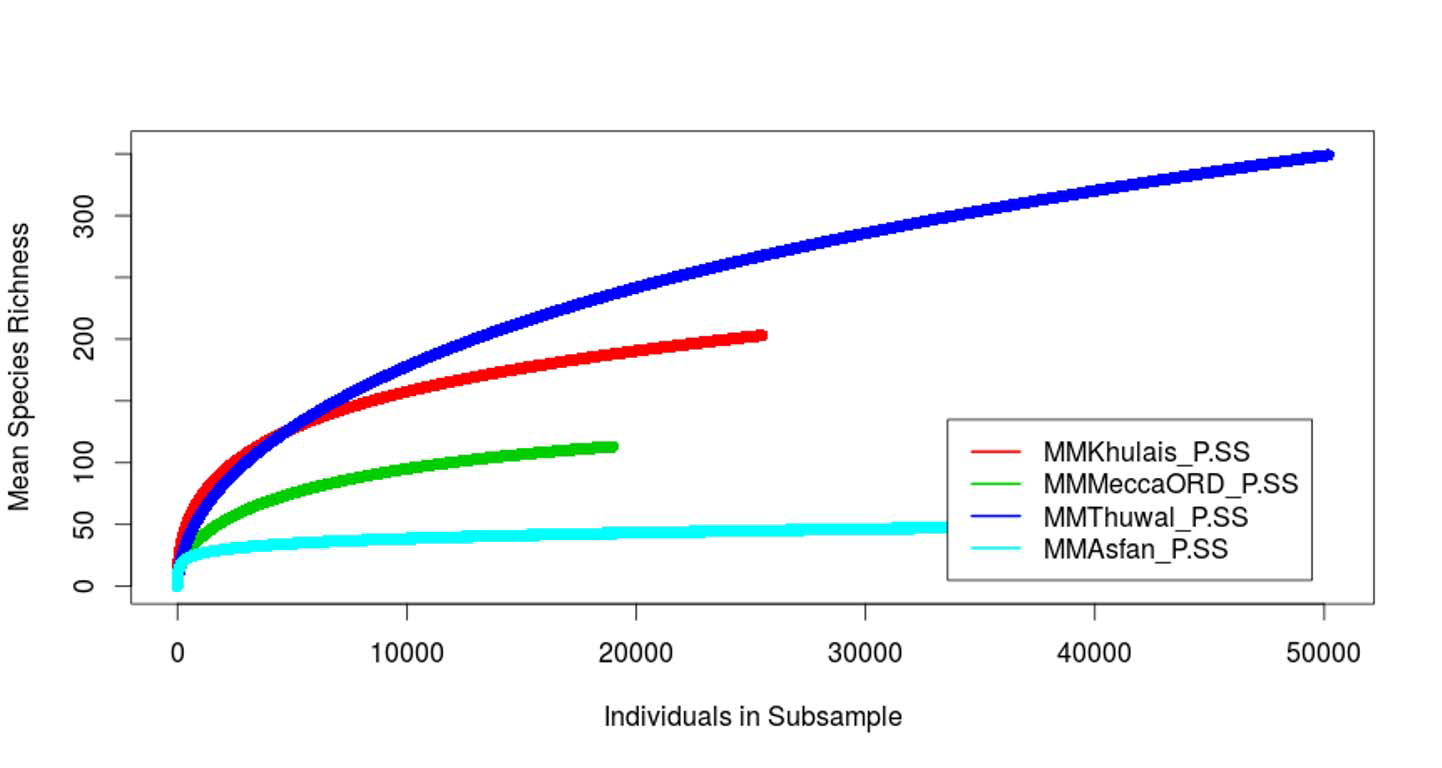

Supplement: S1 Fig — (TIF) [file pone.0185096.s001.tif]
